# Investigation of the mechanism of the anti-cancer effects of *Astragalus propinquus* Schischkin *and Pinellia pedatisecta* Schott (A&P) on melanoma *via* network pharmacology and experimental verification

**DOI:** 10.3389/fphar.2022.895738

**Published:** 2022-08-12

**Authors:** Fang Wang, Juan Bai, Feng Li, Jing Liu, Yanli Wang, Ning Li, Yaqi Wang, Jin Xu, Wanbao Liu, Liting Xu, Lin Chen

**Affiliations:** ^1^ Department of Pharmacy, Xi’an International Medical Center Hospital, Xi’an, Shaanxi Province, China; ^2^ Department of Plastic Surgery, Xijing Hospital, Fourth Military Medical University, Xi’an, Shaanxi Province, China

**Keywords:** *Astragalus propinquus* Schischkin, *Pinellia pedatisecta* Schott, melanoma, PI3K/Akt pathway, network pharmacology

## Abstract

Melanoma is a commonly malignant cutaneous tumor in China. *Astragalus propinquus* Schischkin *and Pinellia pedatisecta* Schott (A&P) have been clinically used as adjunctive drugs in the treatment of malignant melanoma. However, the effect and mechanism of A&P on melanoma have yet to be explored. The current investigation seeks to characterize the active components of A&P and their potential roles in treating malignant melanoma using network pharmacology and *in vitro* and *in vivo* experiments. We first used the traditional Chinese medicine systems pharmacology (TCMSP) database and high-performance liquid chromatography-mass spectrometry (HPLC-MS/MS) to identify a total of 13 effective compounds within A&P. 70 common genes were obtained by matching 487 potential genes of A&P with 464 melanoma-related genes, and then we built up protein-protein interaction (PPI) network of these 70 genes, followed by Gene Ontology (GO) and Kyoto Encyclopedia of Genes and Genomes (KEGG) pathway enrichment analyses. The results revealed that A&P might influence the pathobiology of melanoma through the PI3K/Akt pathway. Molecular docking also confirmed that higher content of ingredients in A&P, including hederagenin, quercetin, beta-sitosterol and stigmasterol, had a strong binding activity (affinity < −5 kcal/mol) with the core targets AKT1, MAPK3 and ESR1. Furthermore, we confirmed A&P could inhibit melanoma cells proliferation and induce cells apoptosis through suppressing the PI3K/Akt signaling pathway by *in vitro* and *in vivo* xenograft model experiments. These findings indicate that A&P may function as a useful therapy for melanoma through the PI3K/Akt pathway.

## Introduction

Melanoma arises from skin neural crest-derived pigmented melanocytes. This aggressive tumor is responsible for approximately 80% of all skin cancer-related mortality, with less than 5% of patients surviving beyond 5 years (Liu et al., 2014; Song et al., 2021). Epidemiological data suggest that high levels of nevus, UV radiation and genetic factors all play a decisive role in the risk for melanoma (Grimaldi et al., 2014). Clinically, conventional therapies for melanoma are restricted to surgical resection, chemotherapy, and radiation, but the prognosis is still not optimistic (Kruijff and Hoekstra, 2012). Currently, the treatments of melanoma is being shaped around targeted therapies for specific melanoma subgroups (Moschos and Pinnamaneni, 2015). While the clinical benefits of targeted therapies are certain, future advances will be only possible if we better understand both the pathogenesis of melanoma and its molecular mechanism of targeted therapies (Kakavand et al., 2016). Melanoma patients would benefit tremendously from novel and effective treatments (Goffin et al., 2005). Recent evidence highlights the potential of traditional Chinese medicine (TCM) as an agent that enhances the chemotherapeutic effect while also preventing the metastatic melanoma (Nobili et al., 2009; Chinembiri et al., 2014). Molecular biology studies reveal that TCM may offer several anti-tumor effects (Qiu and Jia, 2014). Previous studies showed that *Astragalus propinquus* Schischkin (HuangQi in Chinese) and *Pinellia pedatisecta* Schott (BanXia in Chinese). Both *Astragalus propinquus Schischkin and Pinellia pedatisecta* Schott have been shown to significantly reduce chemotherapy toxicity and side effects and may improve patient immunity in those with malignant melanoma (You, 2006). Both herbs were first documented in Shennong’s Classic of Materia Medica. Ancient Chinese medical books recorded that the main effects of *Astragalus propinquus* Schischkin were to replenish Qi and elevate Yang, to strengthen the exterior and reduce sweat, and to eliminate toxins and engender flesh. Currently, the active ingredients of *Astragalus propinquus* Schischkin, in combination with chemotherapy, can promote anti-tumor effect, in addition to reduce complications and side effects induced by routine chemotherapy treatment (Li et al., 2020). *Astragalus* injection induces apoptosis of A375 human malignant melanoma cells by influencing the caspase-3 protein-independent pathway (Wang et al., 2015). *Pinellia pedatisecta* Schott, on the other hand, was revealed to dry dampness, reduce phlegm, reduce nausea, dissolve lumps, and resolve masses. Recent pharmacological studies depict *Pinellia pedatisecta* Schott may be an effective immunomodulating drug for anti-cancer treatment (Wang K. et al., 2021). However, the effective components of *Astragalus propinquus Schischkin and Pinellia pedatisecta* Schott (A&P) and the molecular mechanism of that need to be investigated.

With the development of bioinformatics, network pharmacology represents a method that systematically and comprehensively analyzes drug-target protein based interventions and influences based on the “disease-genes-drug” network (Luo et al., 2020). Network pharmacology has been successfully applied to uncover the pharmacological effect of TCM in the treatment of several diseases, including cancer (Su et al., 2019), cardiovascular disease (Zheng et al., 2014) and stroke (Zhang et al., 2014). A comprehensive drug-target interaction network that depicts core molecules and pathways is easily constructed using network pharmacology.

It is generally known that the PI3K/Akt signaling pathway is a primary intracellular signaling cascade that controls malignant cell migration, apoptosis, and proliferation (Wu C. et al., 2020). Most studies report increased activity of the PI3K/Akt signaling pathway in melanoma cells, with its inhibition being able to reduce melanoma cell proliferation and induce apoptosis (Wu et al., 2020; Long and Pi, 2020; Yan et al., 2020). Akt also referred to as protein kinase B (PKB), is a significant downstream effector of PI3K. Akt plays an antiapoptotic role by phosphorylating target proteins through multiple downstream pathways (Backman and Danielson, 2013; Li et al., 2018). Bcl-2 family proteins are key downstream targets of the PI3K/Akt signaling pathway and are located on the mitochondrial membrane, such as proapoptotic proteins, including Bad, Bax, along with antiapoptotic proteins, such as Bcl-xL and Bcl-2 (Liu et al., 2020). Numerous studies have found that abnormal PI3K/Akt signaling pathway activity contributes to the upregulation of Bcl-2 expression, leading to apoptosis-mediated multidrug resistance against several cancer therapies (Liu et al., 2018). Therefore, inhibition of PI3K/Akt may be an effective anti-melanoma approach.

**FIGURE 1:**
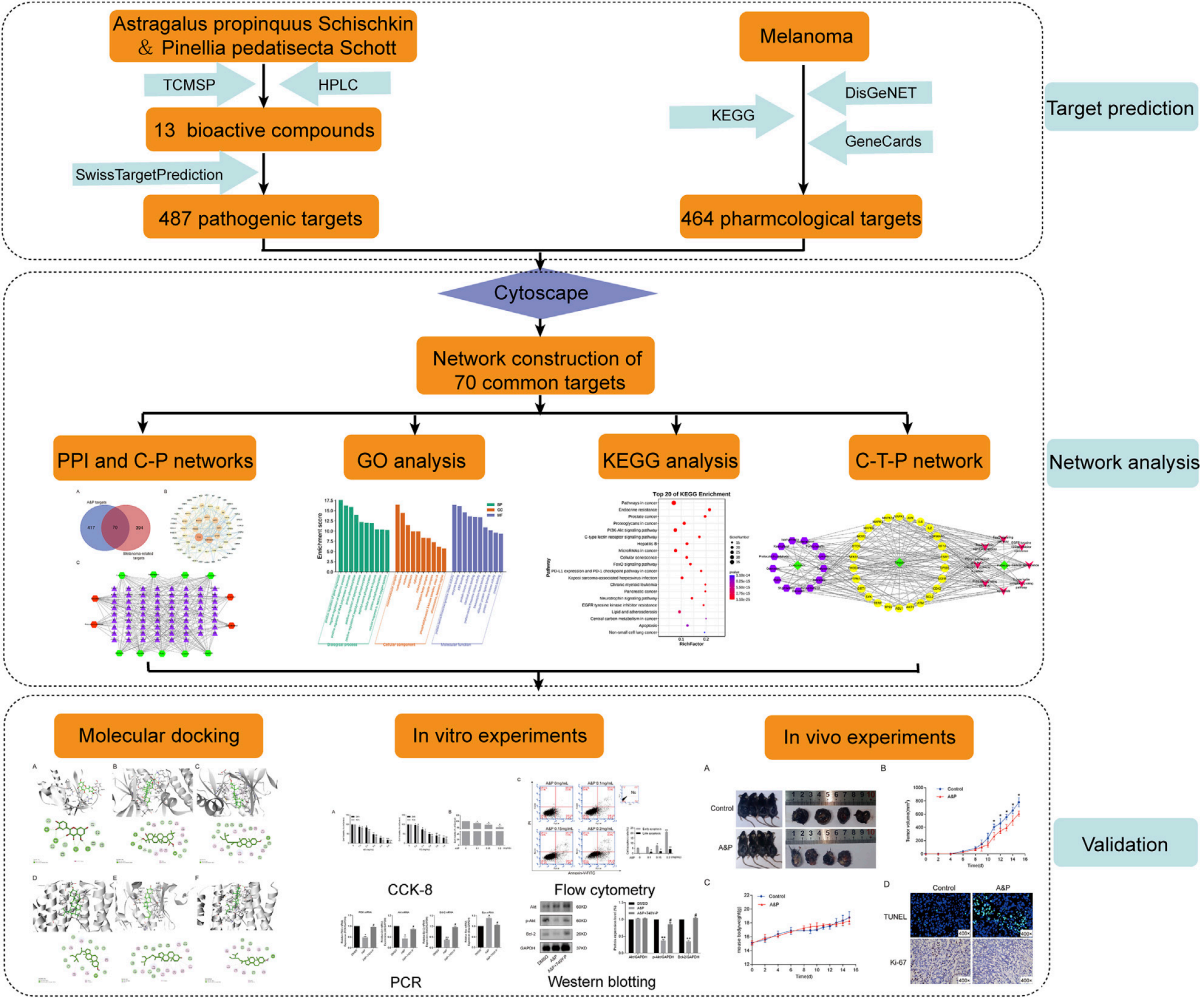
The workflow of this study based on network pharmacology for A&P in treating melanoma.

In this study, we predicted potential targets of A&P and explored the mechanism of A&P treatment for melanoma by network pharmacology analysis. *In vivo* and *in vitro* experiments were performed to provide scientific evidence of the anti-melanoma effect of A&P. The workflow of A&P in treating melanoma was provided in Figure 1.

## Materials and methods

### Preparation of A&P

Two Chinese herbal medicines (*Astragalus propinquus* Schischkin, *Pinellia pedatisecta* Schott) were purchased from Xi’an International Medical Center Hospital (Xi’an, China). *Astragalus propinquus* Schischkin (batch number:1912003) and *Pinellia pedatisecta* Schott (batch number: 191112) were extracted separately. *Astragalus propinquus* Schischkin was extracted as follows: 0.5 kg of finely minced and dried roots of *Astragalus propinquus* Schischkin was extracted using 70% ethanol twice at 2 h each time in each experiment. Low-pressure drying methods were then used to process the extracts, which yielded 189 g of powdery extract. The powder was then dissolved in 70% ethanol and dried under low pressure to yield a total of 152.7 g of dry powder. The *Astragalus propinquus* Schischkin extract (AE) was stored at 4°C. *Pinellia pedatisecta* Schott was extracted as follows: 0.5 kg of dried stems of *Pinellia pedatisecta* Schott was powdered and then extracted using 70% ethanol for 2 h. The extract was filtered and lyophilized, which yielded 10.74 g of dry powder. *Pinellia pedatisecta* Schott extract (PE) was stored at 4°C.

### Identification of the main components in A&P

The main components from A&P were characterized using HLPC-MS/MS analysis, configured with Waters BEH C18 Column (250 × 2.1 mm, 1.7 μm) maintained at 35°C. The mobile phase was constituted of 0.1% methanoic acid in water (A) and acetonitrile (B), using an optimal gradient program as previously established: 0–15 min, 95% (A)-5% (B); 15 min–25 min, 50% (A)-50% (B); 25 min–30 min, 5% (A)-95% (B); 30 min–35 min, 95% (A)-5% (B). The flow rate was adjusted to 0.3 ml/min, while analysis time of each component was 15 min. Mass spectrometry conditions: the positive and negative ionization modes of electrospray ionization (ESI) were used for detection. ESI Source conditions were as follows: Ion Source Gas1(Gas 1): 50, Ion Source Gas2(Gas 2): 50, Curtain Gas (CUR): 25, Source Tempreture: 500°C (positive ion) and 450°C (negative ion), Ion Sapary Voltage Floating (ISVF) 5500 V (positive ion) and 4400 V (negative ion), TOF MS scan range: 100–1,200 Da, product ion scan range: 50–1,000 Da, TOF MS scan accumulation time 0.2 s, product ion scan accumulation time 0.1 s. Second order mass spectrometry was obtained by Information Dependent Acquisition (IDA) and had a high-sensitivity mode. Declustering potential (DP): ±60 V, Collision Energy: 35 ± 15 eV.

### Target prediction of active compounds in A&P

The TCMSP database (http://tcmspw.com/) was used to retrieve pharmacological information of the compounds using “HuangQi” and “BanXia” as keywords. Two key ADME parameters, which were drug similarity (DL ≥ 0.18) and oral bioavailability (OB ≥ 30%), were then selected for the preliminary screening of the collected compounds (Pang et al., 2018). OB refers to the amount of a drug that actually enters circulation within the body after being ingested, while DL represents the similarity between the constituent and an established medication (Ru et al., 2014). A&P active compound targets were explored with the SwissTargetPrediction target prediction platform (http://www.swisstargetprediction.ch/).

### Collection of melanoma-related genes

The melanoma-related genes were extracted from three databases including DisGeNET Database (http://www.disgenet.org/), KEGG (https://www.kegg.jp/) and GeneCards Database (https://www.genecards.org/). The common target genes were screened out as the targets of A&P in the treatment of melanoma.

### PPI network construction and C-T network construction of A&P with melanoma

The A&P-related targets and the melanoma-related targets were intersected, and a Venn diagram of the cross-common targets was obtained. The Cytoscape software (v3.2.1) was used to create a PPI network of A&P with melanoma. To understand the complicated interactions between compounds and their corresponding common targets, the C-T network was constructed and displayed by utilizing the Cytoscape software (v3.2.1). The compounds and targets are represented by nodes and are linked with edges.

### Functional enrichment analyses and construction of the C-T-P network

GO and KEGG functional enrichment analyses were carried out with the DAVID database (https://david.ncifcrf.gov/). Relevant pathway analyses with *p* values < 0.05 were interpreted to be significant and interesting. The Omicshare platform was used to visualize the results of GO and KEGG enrichment analyses. To visually elucidate the mechanisms of A&P in the treatment of melanoma, a network connecting components, core targets and pathways (C-T-P network) was established.

### Molecular docking verification

Among the compounds of A&P, the first 4 compounds with the higher concentration were used as ligands and their 2D structure were downloaded from PubChem (https://pubchem.ncbi.nlm.nih.gov/). Genes corresponding to these four compounds in the top eight core targets of PPI key protein “degree” value were selected as receptors and their 3D structures were obtained from the RCSB PDB database (https://www.rcsb.org/), including AKT1(PDB ID: 1UNQ), MAPK3 (PDB ID: 4QTB) and ESR1 (PDB ID: 7BAA). The protein was imported into AutoDockTools software for format conversion, and all water molecules and small molecule ligands of crystal molecules were removed, polar hydrogen was added, charge was loaded, and active pockets were constructed. AutoDock4 software was used for molecular docking with effective active ingredients, and the binding energy was less than “−5 kcal/mol,” it showed that the target had certain binding activity with the compound (Gaillard, 2018). Finally, Discovery Studio Visualizer software was used to analyze and visualize the 3D and 2D interaction diagrams.

### Cell line and cell culture

Mouse melanoma B16F10 cells were procured from the American Type Culture Collection (ATCC, Manassas, VA, United States). The cells were cultured in high glucose Dulbecco’s modified Eagle medium (DMEM) containing 10% fetal bovine serum (HyClone, Logan, Utah, United States), 100 U/ml each of streptomycin and penicillin (Gibco, Grand Island, NY, United States) and were incubated at 37°C with 5% CO_2_. The cells were then allowed to achieve 70–80% confluency before being exposed to 0.05% trypsin-EDTA and seeded onto 6- or 96-well plates (Corning, NY, United States).

### Cell viability assay

A CCK-8 assay (Beyotime, Shanghai, China) was used to evaluate cell viability. The cells (1 × 10^3^ per well) were seeded onto 96-well plates and stored in DMEM until adherent to the flask wall. The cells were then exposed to either 1.0, 2.0, 3.0, 4.0, and 5.0 mg/ml AE or 0.2, 0.4, 0.6, 0.8, and 1.0 mg/ml PE for 24 and 48 h respectively. After the IC50 was calculated, AE and PE were combined into the wells in different concentrations and proportions for 24 h within the range of no cytotoxicity. At the end of each dose, 10 μl of CCK-8 was added and incubated for 4 h and the absorbance was recorded with a microplate reader at 450 nm (Epoch, Bio Tek, United States).

### Cell apoptosis assay

After a 24-h treatment period using various concentrations and A&P proportions, the concentration ratio of PE and AE was determined to be 1:2. To examine cell apoptosis, cultured B16F10 cells were exposed for 24 h to A&P, rinsed with phosphate-buffered saline, and treated with the Annexin V-FITC Apoptosis Staining/Detection Kit (Beyotime, Shanghai, China). Flow cytometry analysis was conducted with flow cytometry (BD, San Jose, CA, United States).

### RNA extraction and quantitative real-time PCR

B16F10 cells were seeded in 6-well plates and incubated for 24 h at 37°C. Cells were treated with A&P (PE:AE = 1:2, the concentration of PE was 0.2 mg/ml) for 24 h. DMSO (0.1%) was used as the drug vehicle. For experimental groups, cells were exposed to A&P alone or in combination with 20 μM 740Y-P (PI3K/Akt pathway activator; MedChemExpress, China) for 24 h. Total RNA were extracted from B16F10 cells by the TRIzol reagent (Invitrogen, Carlsbad, CA, United States) in strict compliance with manufacturer instructions. RNA was reverse-converted into cDNA and subsequently amplified with the aid of a reverse transcription kit (RiboBio, Guangzhou, China) in accordance with the instructions. All primers are as follows: GAPDH (Forward: AAT​GGA​TTT​GGA​CGC​ATT​GGT, Reverse: TTT​GCA​CTG​GTA​CGT​GTT​GAT), PI3K (Forward: CGA​GAG​TGT​CGT​CAC​AGT​GTC, Reverse: TGT​TCG​CTT​CCA​CAA​ACA​CAG), Akt (Forward: CCC​TGC​TCC​TAG​TCC​ACC​A, Reverse: TGT​CTC​TGT​TTC​AGT​GGG​CTC), Bcl-2 (Forward: GAG​CCT​GTG​AGA​GAC​GTG​G, Reverse: CGA​GTC​TGT​GTA​TAG​CAA​TCC​CA) and Bax (Forward: AGA​CAG​GGG​CCT​TTT​TGC​TAC, Reverse: AAT​TCG​CCG​GAG​ACA​CTC​G). Quantitative Real-Time PCR (qRT-PCR) was carried out using the Applied Biosystems StepOnePlus Real-Time PCR System (Applied Biosystems, Singapore).

### Western blot analysis

After treatment as above, total proteins were extracted from cells using a Cell Lysis Buffer (Beyotime, Shanghai, China). A BCA protein detection reagent (Beyotime, Shanghai, China) allowed for the evaluation of protein concentration. A 10% SDS-PAGE gel was used to separate the proteins into their components, followed by immunoblotting the proteins onto PVDF membranes. 5% milk in PBS plus 0.1% Tween 20 was used to block endogenous reactions for an hour. This was followed by overnight incubation of the membranes with the primary antibodies: Akt (#9272, 1:1,000), p-Akt (#13038, 1:1,000), Bcl-2 (#3498, 1:1,000) and GAPDH (#5174, 1:1,000) at 4°C. And then the membranes were incubated for 2 h at room temperature with HRP-conjugated secondary antibodies (Goat anti-rabbit IgG, 1:1,000). Finally, the bands were imaged using an enhanced chemiluminescence system (ECL, ZETA, Beijing, China). The protein band gray values were calculated using the ImageJ software. Each experiment was repeated three times.

### Tumor xenograft model *in vivo*

A total of 16 male C57BL/6 mice (6–8 weeks old, 15.15 ± 0.29 g) were purchased from ChengDu Dossy Experimental Animals Co. Ltd (Chengdu, China). The mice were kept in a temperature-controlled chamber under a 12-h light-dark cycle with free access to food and water. Accurately 0.1 ml B16F10 cells (3 × 10^6^ cells per mouse) were subcutaneously injected into the right lower back region of mice. The mice weight and tumour length and width were measured every 2 days. The tumor volume was calculated using the following formula: tumor volume (mm^3^) = 1/2 × length × width^2^ (mm^3^). When the tumor volume reached about 100 mm^3^, the mice were devided randomly into two groups: the A&P-treated group and control group (*n* = 4 per group). The A&P-treated group was intragastrically administered with A&P (25 mg/kg PE and 50 mg/kg AE) per day. Control group received the same dose of phosphate buffer saline. Data measurement was also taken per day. The mice were sacrificed by cervical dislocation after 15 days of treatment, and the tumors were harvested for the subsequent testing. All animal experiments were approved by the Ethics Committee of Xi’an International Medical Center Hospital.

### TdT-UTP nick end labeling and immunocytochemistry

Frozen section samples of tumor tissues were prepared, cut into 5 μm-thick and then were immersed in 4% paraformaldehyde. For TUNEL staining, sections were digested with proteinase K for 25 min at 37°C and then incubated with the TUNEL reaction mixture (Roche) for 1 h in a dark-wet box at 37°C. For Ki-67 staining, the slices were immersed in 0.01 M citrate buffer (PH 6.0) and placed in the microwave oven on high heat until boiling and power off, repeated after an interval of 5 min. After washed two times with PBS, samples were incubated with Ki-67 antibody (abcam; 1:250) overnight at 4°C and then incubated with secondary antibody at 37°C for 30 min. Finally, the sections were observed and photographed under a microscope (Motic).

### Statistical analysis

GraphPad Prism 6.02 (San Diego, CA, United States) was used to perform all statistical analyses, will all findings depicted in terms of mean ± SD. The Student’s t-test or two-way analysis of variance (ANOVA) was used to analyze data. *p* < 0.05 was considered to be significant.

## Results

### Identification of active compounds of *Astragalus propinquus*

**FIGURE 2:**
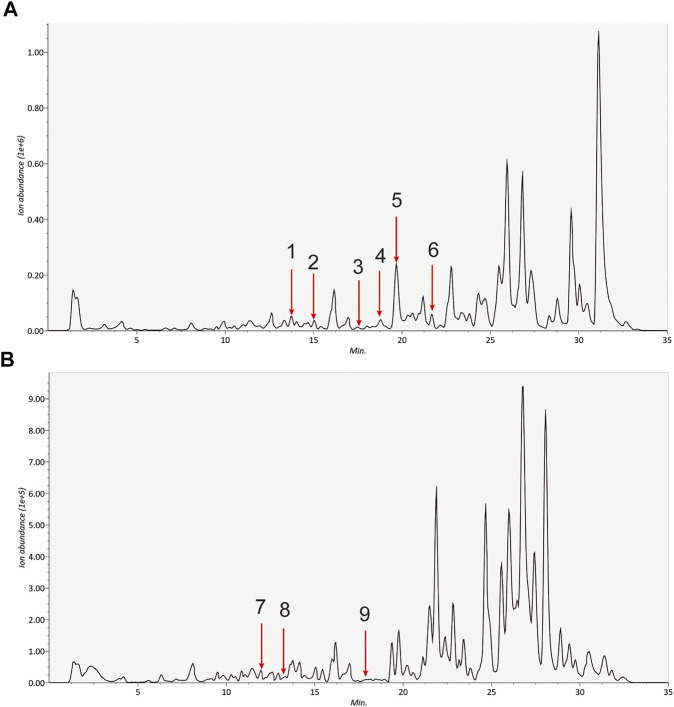
Identification of the detected componds in the extracts of A&P by HPLC-MS/MS. **(A)** Positive ion **(B)** Negative ion. The number corresponds to the compound: 1. Calycosin; 2. Formononetin; 3. Kaempferol; 4. Beta-sitosterol; 5. Hederagenin; 6. Stigmasterol; 7. Quercetin; 8. Isorhamnetin; 9. Baicalein.

HPLC-MS/MS was adopted to identified the main medicinal components in A&P. According to the chromatograms, a total of nine chemical constituents were identified (Figure 2). The concentrations of the compounds were listed in Supplementary Table S1. In addition to them, bifendate was hardly detected by HPLC-MS/MS, but it had 27 common targets, the largest number, with melanoma-related pharmacological targets. And it had an anti-cancer effect against multidrug-resistant tumors (Ren et al., 2016). So bifendate was also considered as a candidate component. To retrieve the active components of A&P, two key ADME parameters, DL (≥0.18) and OB (≥30%) were selected for the preliminary screening of the collected compounds. Although some ingredients do not meet the screening criteria, they have clinical efficacy; We also retained these for a full analysis of the components that we studied. For example, OB of rutin was very low and coumarin has a low DL, but they are the major components of *Astragalus propinquus Schischkin,* which had cytotoxic effects in melanoma cells (Bhattarai et al., 2021; Pinzaru et al., 2021). Another component, protocatechualdehyde, an active constituent from *Pinellia pedatisecta* Schott*,* has a low DL, but it induces cell apoptosis in melanoma B16-F10 cells (Zhong et al., 2020). Therefore, they were also included as candidate components. Hence, a total of 13 ingredients were selected as active ingredients in A&P as shown in Table 1. Specific information of these components are provided in Supplementary Table S2.

**TABLE 1:** A total of 13 ingredients in A&P.

NO	Molecule name	CAS	Herb
1	Quercetin	73123-10-1	*Astragalus propinquus* Schischkin
2	Hederagenin	465-99-6	*Astragalus propinquus* Schischkin
3	Isorhamnetin	480-19-3	*Astragalus propinquus* Schischkin
4	Bifendate	73536-69-3	*Astragalus propinquus* Schischkin
5	Formononetin	485-72-3	*Astragalus propinquus* Schischkin
6	Calycosin	20575-57-9	*Astragalus propinquus* Schischkin
7	Kaempferol	520-18-3	*Astragalus propinquus* Schischkin
8	Rutin	115888-40-9	*Astragalus propinquus* Schischkin
9	Coumarin	91-64-5	*Astragalus propinquus* Schischkin
10	Baicalein	491-67-8	*Pinellia pedatisecta* Schott
11	Beta-sitosterol	83-46-5	*Pinellia pedatisecta* Schott
12	Stigmasterol	83-48-7	*Pinellia pedatisecta* Schott
13	Protocatechualdehyde	139-85-5	*Pinellia pedatisecta* Schott

### Analyses of PPI and C-T networks

**FIGURE 3:**
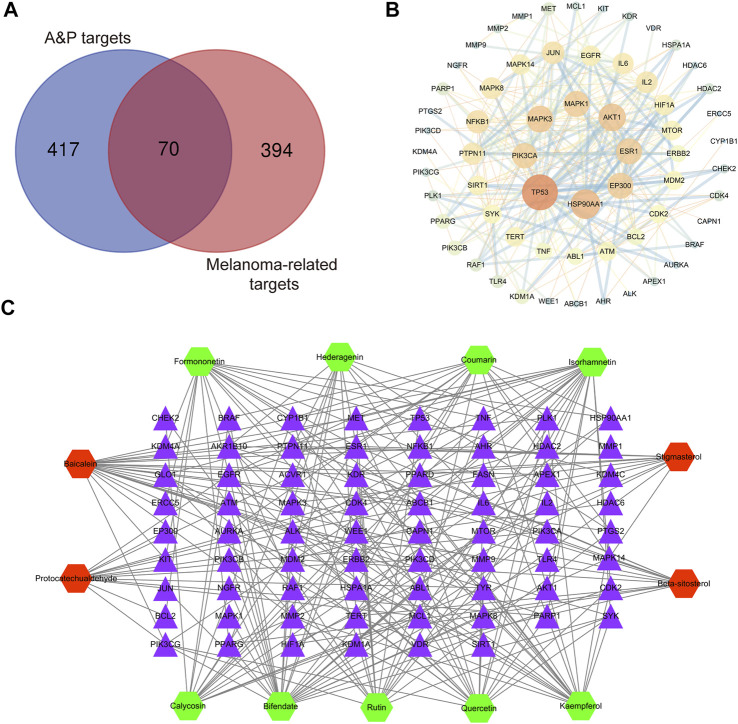
Network construction. **(A)** Venn diagram of candidate targets in A&P and melanoma. **(B)** PPI network. Nodes represent relevant targets; edges represent interactions between. **(C)** C-T network. The green hexagonal nodes represent the compounds of *Astragalus propinquus* *Schischkin*, the red hexagonal nodes represent the compounds of *Pinellia pedatisecta* Schott. The purple triangular nodes represent the intersection targets between A&P and melanoma.

A total of 487 pathogenic targets were found to be associated with the compounds of A&P from the TCMSP (Supplementary Table S3). 464 melanoma-related pharmacological targets were screened out from DisGeNET Database and KEGG Platform (Supplementary Table S4). 70 common targets were determined to function as potential A&P targets in melanoma treatment (Figure 3A). Specific information of the 70 common targets are described in Supplementary Table S5. Data from 70 common targets were incorporated into STRING to build a PPI network to explore the interactive relationships with each other. And then, the PPI network was optimized by Cytoscape v3.2.1 (Figure 3B). In the PPI network, the nodes with higher degree might be more important in melanoma treatment. The detailed information of the PPI network are presented in Supplementary Table S6. 26 core targets with degree values above the median were identified. Among them, the top eight targets were tumor protein p53 (TP53), heat shock protein 90 alpha family class A member 1 (HSP90AA1), AKT serine/threonine kinase 1 (AKT1), mitogen-activated protein kinase 3 (MAPK3), E1A binding protein p300 (EP300), estrogen receptor 1 (ESR1), mitogen-activated protein kinase 1 (MAPK1), phosphatidylinositol-4,5-bisphosphate 3-kinase catalytic subunit alpha (PIK3CA).

Based on the 13 compounds and 70 intersection targets, we constructed a C-T network by Cytoscape v3.2.1. As shown in Figure 3C, 13 hexagonal nodes represent the active compounds of A&P, and 70 triangular nodes represent the common targets of A&P in melanoma treatment. The network showed the interrelation between the compounds of A&P and the target genes. The detailed information of the network are displayed in Supplementary Table S7. The compounds with higher degree of connections were bifendate, baicalein, isorhamnetin and quercetin. For the target analysis, ESR1 was linked to the largest number compounds. In addition, AKT1, HSP90AA1 and MAPK3 were respectively connected to four compounds. These findings suggest that A&P treats melanoma with multiple compounds acting on multiple genes.

### Biological functions and pathway enrichment analyses of A&P-treated melanoma

**FIGURE 4:**
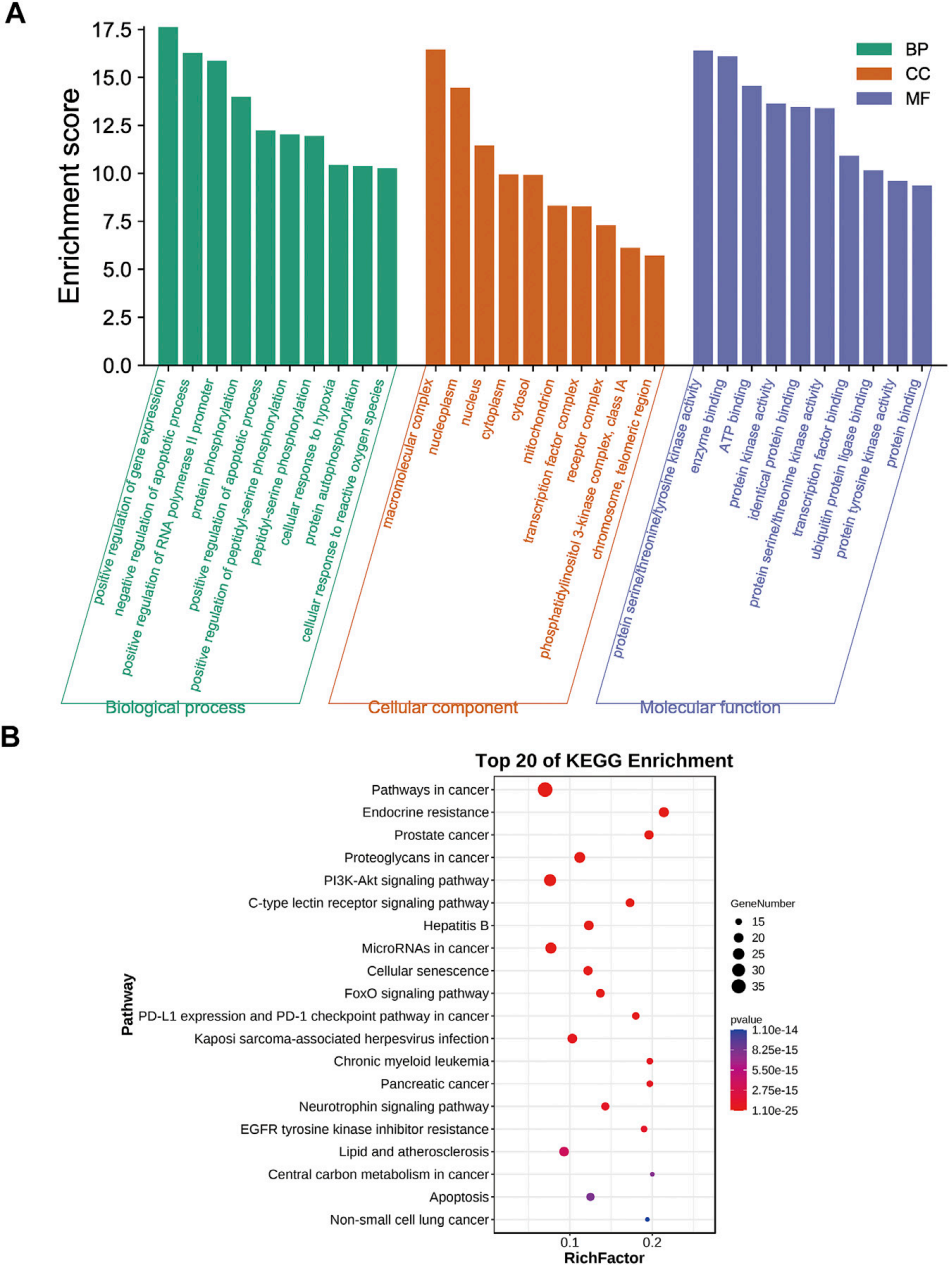
Biological functional enrichment analyses. **(A)** The top 10 of biological processes, cellular components and molecular functions. **(B)** The top 20 of KEGG pathways.

GO enrichment analysis of the 70 potential therapeutic targets was carried out to characterize the candidate biological functions of A&P in treating melanoma, including biological process (BP), cellular component (CC), and molecular function (MF) categories. A total of 408 BPs, 38 CCs, 85 MFs and 152 pathways (*p* < 0.05) were obtained from the DAVID database (Supplementary Table S8). The BP analysis showed the key targets to be significantly correlated with positive regulation of gene expression, negative regulation of apoptotic process, positive regulation of transcription from RNA polymerase II promoter and protein phosphorylation. The CC analysis were associated with macromolecular complex, nucleoplasm, nucleus and cytoplasm. The MP analysis suggested the main targets to be related to protein serine/threonine/tyrosine kinase activity, enzyme binding, ATP binding and protein kinase activity. The top 10 BPs, CCs, and MFs items are shown in Figure 4A. The top 20 potential pathways of KEGG analysis results are presented in Figure 4B. Among them, there were eight signaling pathways, including PI3K-Akt signaling pathway, C-type lectin receptor signaling pathway, Cellular senescence, FoxO signaling pathway, PD-L1 expression and PD-1 checkpoint pathway in cancer, Neurotrophin signaling pathway, EGFR tyrosine kinase inhibitor resistance and apoptosis. The analysis results showed that the PI3K-Akt signaling pathway was the top signaling pathway. This pathway contains many key genes of 26 core targets, including TP53, HSP90AA1, AKT1, EGFR, MAPK1, MAPK3, SYK, IL2, MTOR, NFKB1, IL6, PIK3CA, CDK2, BCL2 and MDM2. These results suggest that the PI3K-Akt signaling pathway may be a vital pathway for A&P treating melanoma.

### Analysis of C-T-P network

**FIGURE 5:**
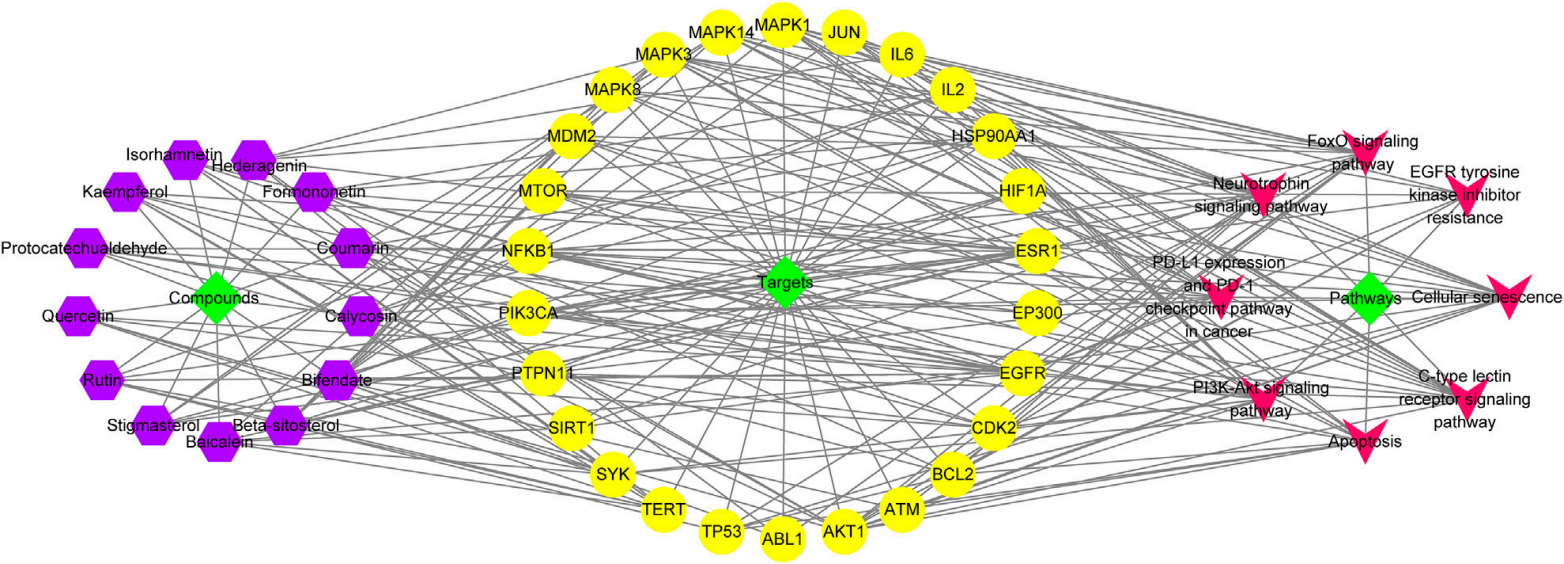
C-T-P network. The orange and green nodes represent the potential active ingredients and their predominant targets of A&P-treating melanoma, while the purple nodes represent the pathways.

**FIGURE 6:**
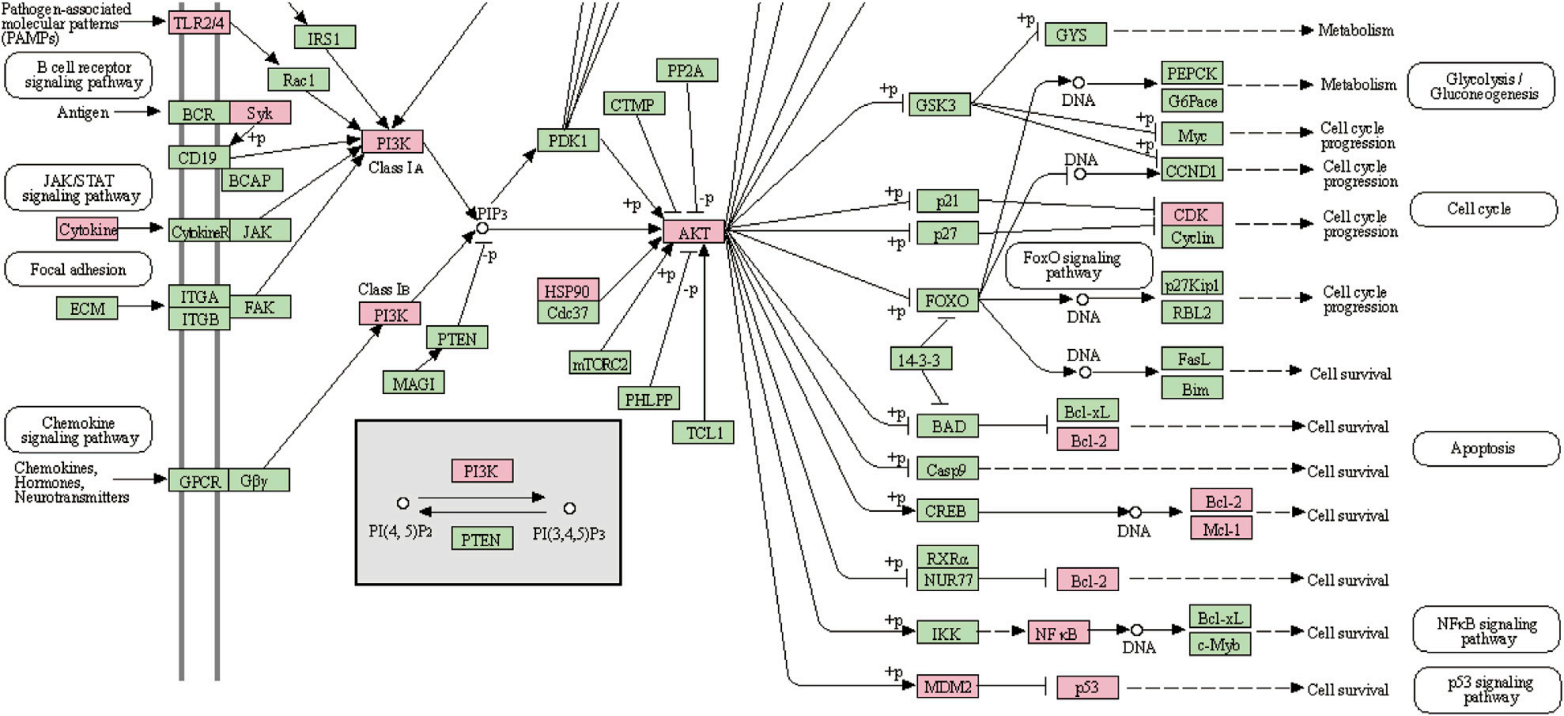
PI3K-Akt signaling pathway. The pink boxes indicate the interactive targets.

We proceeded to establish a C-T-P network constructed from 26 core targets, 13 active compounds and 8 signalling pathways, in order to characterize the therapeutic relationship between A&P and melanoma (Figure 5). Several different targets within these pathways were appeared to be associated with melanoma treatment. This network depicts direct relationships between compounds, targets, and pathways. Purple hexagonal nodes indicate compounds, red angular nodes indicate pathways, yellow round nodes indicate targets, and lines indicate the interactions between them. Additionally, the PI3K-Akt signaling pathway covered the most targets. In addition, we further isolated the network relationships for additional annotation of the KEGG pathway. The network places significant emphasis on the PI3K-Akt signaling pathways (Figure 6). This suggests that A&P may act on melanoma by modulating the PI3K/Akt signaling pathway.

### Molecular docking analysis

**FIGURE 7:**
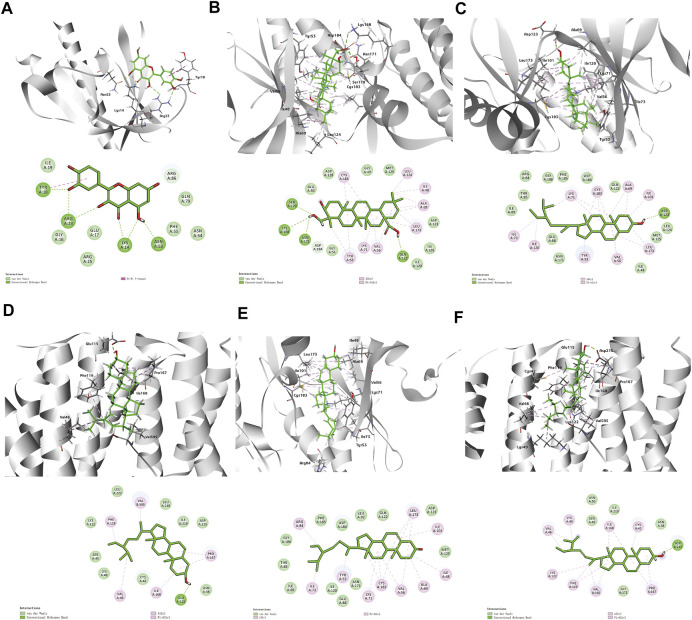
3D and 2D diagrams of binding models. **(A)** Quercetin -AKT1, **(B)** Hederagenin-MAPK3, **(C)** Beta-sitosterol-MAPK3, **(D)** Beta-sitosterol-ESR1Co., **(E)** Stigmasterol-MAPK3, **(F)** Stigmasterol-ESR1.

According to the above results, we selected four compounds of higher content in A&P and their corresponding core targets to do molecular docking, including Quercetin -AKT1, Hederagenin-MAPK3, Beta-sitosterol-MAPK3, Beta-sitosterol-ESR1, Stigmasterol-MAPK3 and Stigmasterol-ESR1. The docking results as follows: Quercetin was docked to AKT1 with four hydrogen bonds LYS14 (2.1 Å), TYR18 (2.4 Å), ARG23 (2.4 Å) and ASN53 (1.8 Å) and by forming hydrophobic interactions with TYR18 (Figure 7A). Hederagenin could bind to MAPK3 by forming hydrophobic interactions with eight residues (ILE48, ALA69, TYR53, VAL56, LYS71, LEU124, LEU173, CYS183) and four hydrogen bonds with GLN122 (2.1 Å), LYS168 (3.1 Å), SER170 (2.2 Å) and ASN171(2.0 Å) (Figure 7B). Beta-sitosterol occupied the hydrophobic pocket composed of the residues (TYR53, VAL56, ALA69, LYS71, ILE73, ILE101, ILE120, LEU173, CYS183) and form a hydrogen bond with ASP123 (1.8 Å) in MAPK3 (Figure 7C), as well as the hydrophobic pocket composed of the residues VAL46, PHE119, PRO167, ILE168 and form a hydrogen bond with GLU115 (1.9 Å) in ESR1 (Figure 7D). Stigmasterol was docked to MAPK3 mainly by hydrophobic interactions with 10 residues (ILE48, TYR53, VAL56, ALA69, LYS71, ILE73, ARG84, ILE101, LEU173, CYS183) (Figure 7E). Stigmasterol bound to ESR1 by forming hydrophobic interactions with seven residues (CYS42, LYS49, VAL46, LYS122, PHE119, PRO167, VAL595) and a hydrogen bond with ASP215 (1.8 Å) (Figure 7F). The results showed that these receptors had strong bindings with the ligands (Table 2). Based on that AKT1, MAPK3 and ESR1 were included in cancer pathways, as well as AKT1 and MAPK3 included in the PI3K-Akt signaling pathway, the docking results again confirmed the prediction of network pharmacology.

**TABLE 2:** Docking scores of between four compounds and their corresponding core targets.

Ligend	Core target	Affinity (kcal/mol)
Quercetin	AKT1	−6.04
Hederagenin	MAPK3	−8.10
Beta-sitosterol	MAPK3	−11.32
Beta-sitosterol	ESR1	−7.03
Stigmasterol	MAPK3	−10.39
Stigmasterol	ESR1	−6.69

### A&P inhibited the proliferation and promoted the apoptosis of B16F10 melanoma cells

**FIGURE 8:**
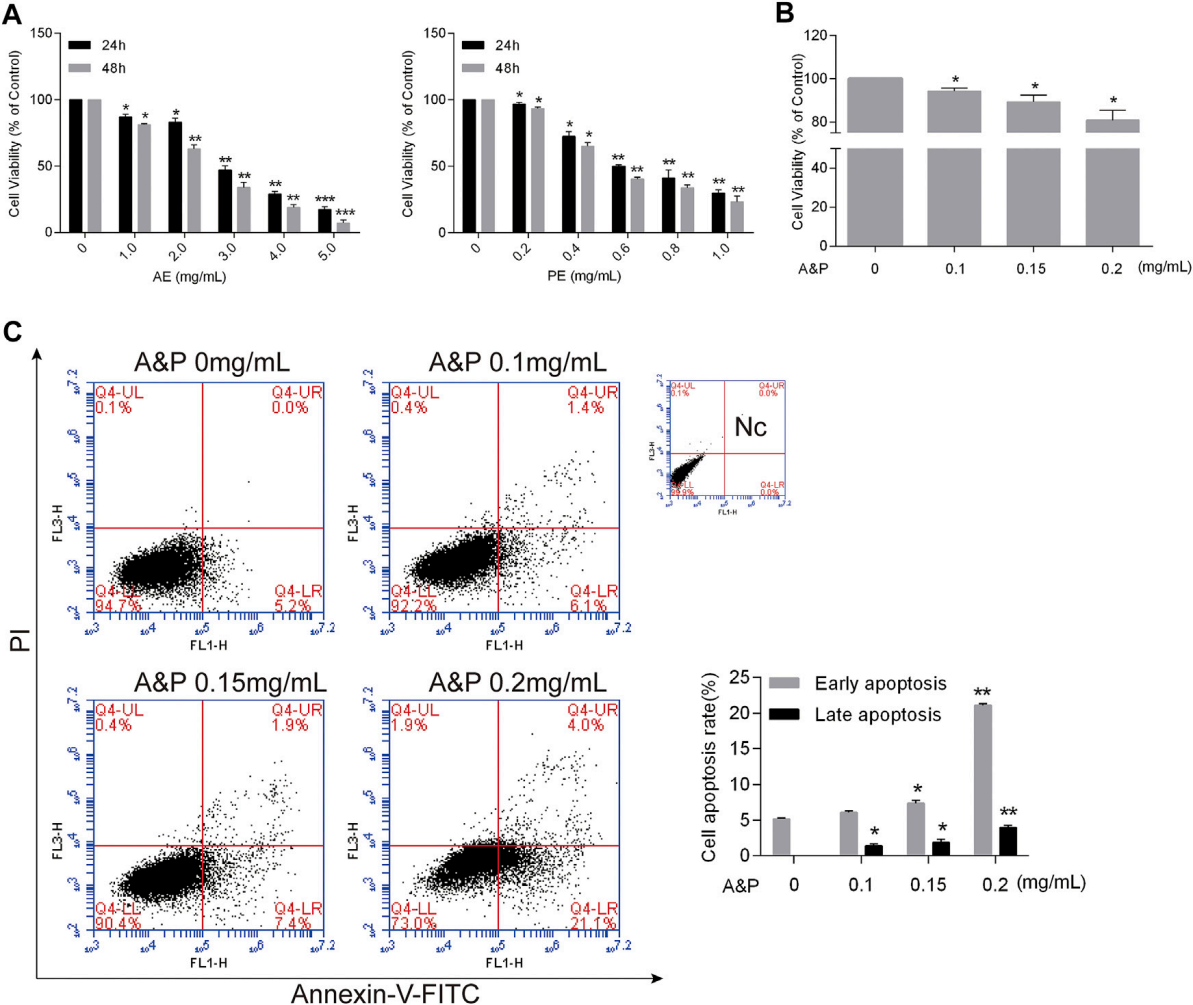
A&P inhibited proliferation and promoted apoptosis by on B16F10 cells. **(A)** CCK-8 assay. Indicated that AE and PE inhibited the proliferation of B16F10 cells in a dose-dependent manner. After 24 and 48 h of treatment. DMSO was used as the control treatment (*n* = 3 per group). **(B)** CCK-8 assay of A&P (PE:AE = 1:2, in a concentration of PE) acted on B16F10 cells (*n* = 3 per group). **(C)** Representative apoptosis analysis indicated A&P promoted the cell apoptosis as shown by flow cytometry assay (*n* = 3 per group). Values are shown as the mean ± SD, *p < 0.05 and **p < 0.01 vs. control group.

Firstly, different concentrations of AE (0–5.0 mg/ml) and PE (0–1.0 mg/ml) were used to treat B16F10 melanoma cells. Both AE and PE remarkably inhibited malignant cell growth in a dose-dependent manner (Figure 8A). Furthermore, the half-maximal inhibitory concentration (IC50) of cells was determined at 24 h. The IC50 of AE on B16F10 melanoma cells was 2.86 ± 0.12 mg/ml, and the IC50 of PE on B16F10 melanoma cells was 0.65 ± 0.23 mg/ml. Based on the IC50 value, compounds PE and AE acted on cells at ratios of 1:1, 1:2, 1:5, and 1:10 (Supplementary Figure S1). The results showed that compound PE and AE at a concentration of 1:2 blocked malignant cell growth in a dose-dependent manner (Figure 8B). Cell apoptosis increased after compound PE and AE treatment. Flow cytometry analysis demonstrated that the early and late apoptosis of B16F10 cells was significantly promoted (Figure 8C) by compound PE and AE treatment.

### Validation of PI3K-Akt as a major target pathway of A&P in melanoma

**FIGURE 9:**
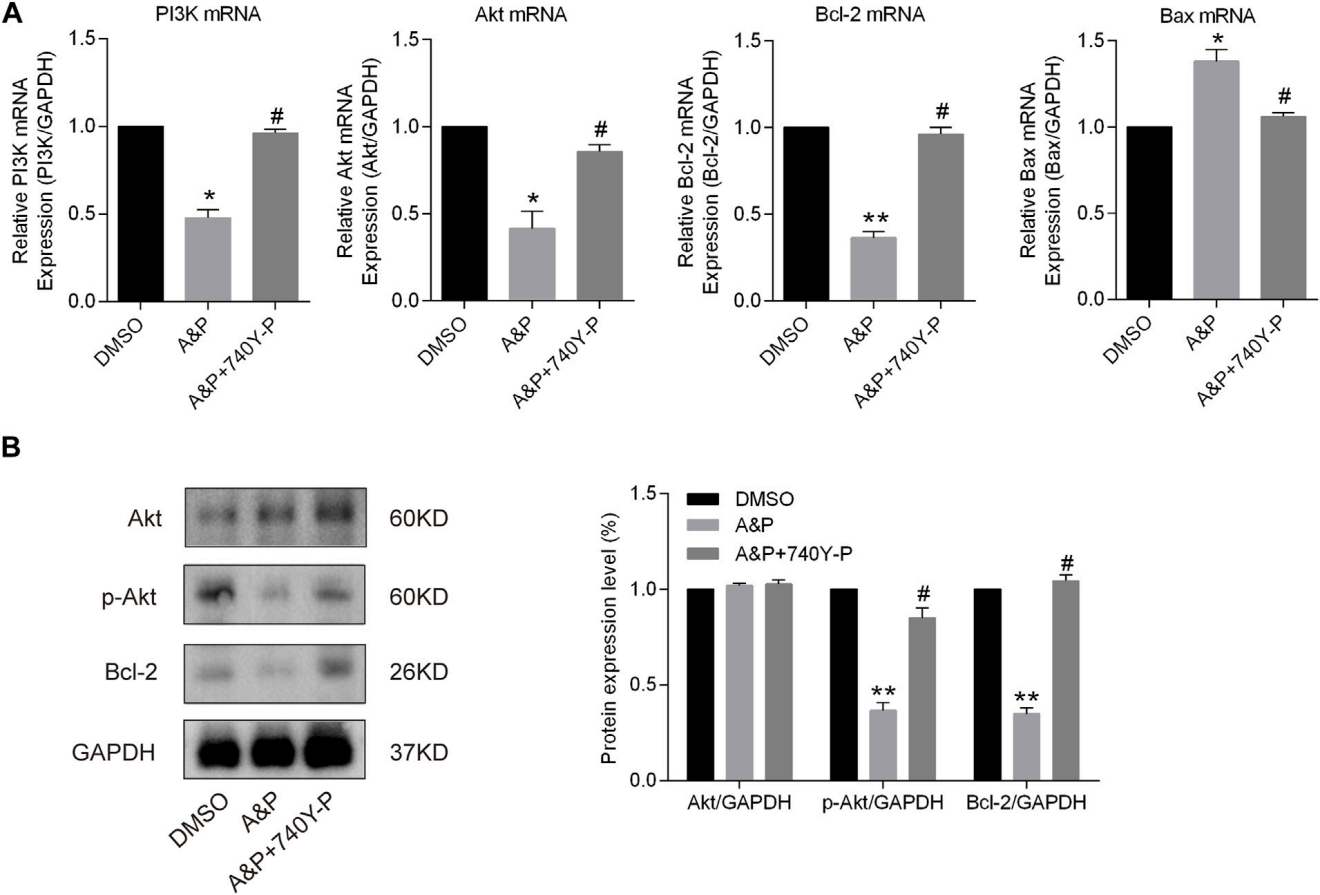
A&P modulated the expression of the PI3K/Akt signaling pathway and Bcl-2 family proteins. B16F10 cells were treated with A&P (PE:AE = 1:2, the concentration of PE was 0.2 mg/ml) alone or in combination with 20 μM 740Y-P for 24 h. **(A)** Relative mRNA expression levels of PI3K, Akt, Bcl-2, and Bax were detected by qRT-PCR analysis (*n* = 3 per group). **(B)** Expression levels of Akt, p-Akt, and Bcl-2 were evaluated by western blot analysis (*n* = 3 per group). Values are shown as the mean ± SD, *p < 0.05 and **p < 0.01 vs. control group.

A&P appears to exert its therapeutic effect on melanoma through 26 active compounds targeting 62 melanoma-associated genes that influence the PI3K/Akt and apoptosis signaling pathways. Cell apoptosis was found to be enhanced by A&P, and we sought to further investigate whether the anti-tumor effects of A&P on B18-F10 cells were mediated by inhibition of the PI3K/Akt pathway. B16F10 cells were treated with A&P (PE:AE = 1:2, the concentration of PE was 0.2 mg/ml) alone or in combination with 20 μM 740Y-P. qRT-PCR analysis demonstrated that A&P suppressed the relative expressions of PI3K, Akt, and Bcl-2 mRNA and promoted the relative expression of Bax mRNA and the addition of 740Y-P could reverse the changes by A&P (Figure 9A). Meanwhile, western blot analysis revealed that the expression of p-Akt and Bcl-2 were reduced in A&P-treated group, but was rised in the A&P + 740Y-P group (Figure 9B). As a whole, these data supported the hypothesis that A&P stimulated B16F10 cell apoptosis by acting on the PI3K/Akt pathway.

### A&P inhibited tumor growth *in vivo*

**FIGURE 10:**
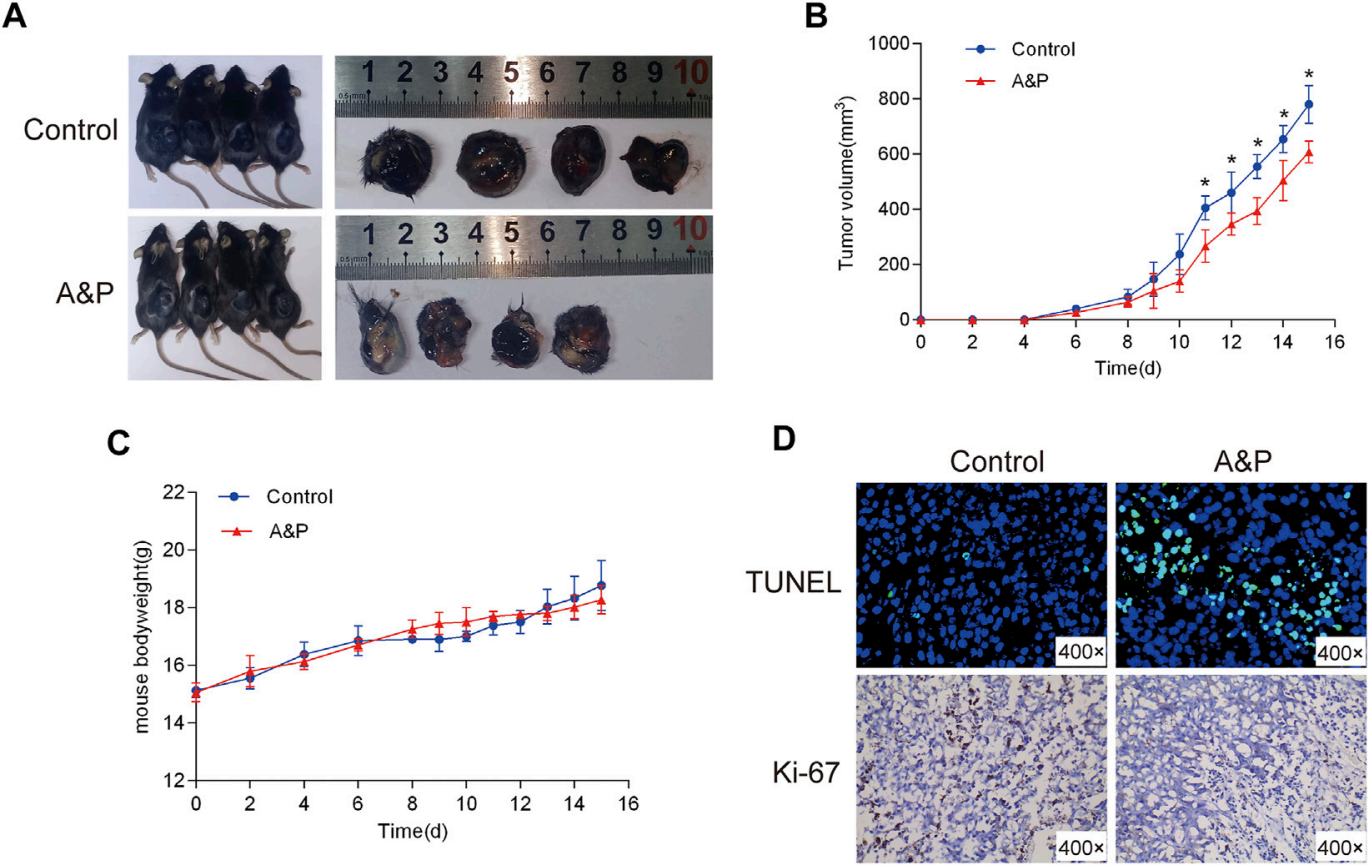
Effects of A&P on B16F10 tumor growth *in vivo* xenograft mice model. **(A)** Gross observation of B16F10 xenograft tumors harvested from the mice demonstrated that A&P inhibited B16F10 xenograft tumor growth (*n* = 4 per group). **(B)** Effects of A&P on tumor volume were recorded at different time points (*n* = 4 per group, *p < 0.05 vs. control group) **(C)** Effects of A&P on bodyweight of the mice were recorded at different time points (*n* = 4 per group). **(D)** The apoptosis and proliferation of tumor tissues were indicated by TUNEL and Ki-67staining (×400 magnification).

As shown in Figure 10A, *In vivo* experiments the B16F10 cell xenograft model was established to further investigate whether A&P could effectively inhibit tumor growth. After 15 days of treatment, the mice were sacrificed. The results showed that the tumor volumes from the A&P-treated group were remarkly smaller in comparison with that in the control group (Figure 10B), while the bodyweights of mice were no significant differences between these two groups (Figure 10C). TUNEL and Ki-67 staining results showed that more apoptic cells and fewer proliferative cells were observed in the A&P-treated group than that in the control group (Figure 10D). These findings demonstrated that A&P suppressed the B16F10 tumor growth *in vivo*.

## Discussion

Increasing evidence indicates that TCM is an effective anti-cancer agent (Xiang et al., 2019; Wang Y. et al., 2021). The current investigation determines the chemotherapeutic qualities of A&P extracts on melanoma. A&P extracts markedly inhibited melanoma *in vivo* and suppressed cell proliferation and augmented melanoma cell apoptosis *in vitro*. Therefore, it is valuable to explore the impact and biological effects of A&P in melanoma.

Network pharmacology is a novel means of exploring the molecular and cellular workings of drugs in a variety of tissue and organism levels (Berger and Iyengar, 2009). Herein, a network pharmacology approach was utilized to uncover the molecular biology of A&P in order to further understand the complementary effects of A&P ingredients. A thorough analysis of the TCMSP database and HPLC-MS/MS revealed a total of 13 active compounds. We then performed PPI network, GO and KEGG pathway analyses to elaborate the mechanism by which A&P suppresses cell proliferation and stimulates apoptosis. A PPI network analysis indicated that eight predominant bio targets of TP53, HSP90AA1, AKT1, MAPK3, ESR1, EP300, MAPK1, and PIK3CA were the most important molecules in A&P and melanoma with the greatest degree scores. GO and KEGG analyses highlighted cancer-related pathways, including the PI3K/Akt signaling pathway, as key roles in tumor development.

In order to further study the interaction between the active components and their corresponding core targets, four components with higher content in HLPC detection were selected for molecular docking with targets. The results confirmed that hederagenin, quercetin, beta-sitosterol and stigmasterol, had a strong binding activity (affinity < −5 kcal/mol) with the core targets AKT1, MAPK3 and ESR1. Among those hub molecules, TP53, HSP90AA1, AKT1, MAPK3 and ESR1 were the primary target nodes that demonstrated the larger degree, suggesting that there was a high probability that these molecules had an important role in the A&P regulatory network. Moreover, pathway enrichment analysis revealed PI3K/Akt signaling to be significantly influenced by TP53, HSP90AA1, AKT1 and MAPK3. The PI3K/Akt signaling pathway is a crucial biological mechanism that impacts cell apoptosis, migration, proliferation, and cell cycle progression (Chen et al., 2016). In the current study, we found that A&P decreased the transcription of PI3K and further inhibited the phosphorylation of Akt at the protein level. Mounting evidence has shown that the PI3K/Akt signaling pathway is critical in mediating tumor growth (Yang et al., 2019; Xu et al., 2020). Studies have shown that TCM intervention lowered p-Akt levels along with the concentration gradient of TCM treatment while maintaining an unchanged total overall Akt level (Gu et al., 2017; Zhao et al., 2018). These findings mirror our results. Our cell experiments confirmed this, where PI3K activator 740Y-P rescued partial inhibition effects of A&P (Figure 9). Finally, we carried out *in vivo* experiments and found that A&P extracts could inhibit the growth of melanoma by TUNEL and Ki-67 staining. These results found that TCM A&P could significantly inhibit melanoma through PI3K/Akt pathway.

## Conclusion

Our study found a combination of network pharmacology and experimental validation to demonstrate the mechanism of A&P for treating melanoma. *In vitro* and *in vivo* experiments confirmed A&P could inhibit melanoma cells proliferation and induce melanoma cells apoptosis through PI3K/Akt signaling pathway. This study not only provides possible theoretical mechanism and experimental evidence for A&P treating melanoma but also may lead to PI3K/Akt-based therapeutic strategies for melanoma.

## Data Availability

The original contributions presented in the study are included in the article/Supplementary Material, further inquiries can be directed to the corresponding author.
