# Supplementary figures and images for: Investigation of the mechanism of the anti-cancer effects of Astragalus propinquus Schischkin and Pinellia pedatisecta Schott (A&P) on melanoma via network pharmacology and experimental verification
Source: Front Pharmacol. 2022 Aug 12;13:895738. doi: 10.3389/fphar.2022.895738 (PMC9411814; doi:10.3389/fphar.2022.895738)

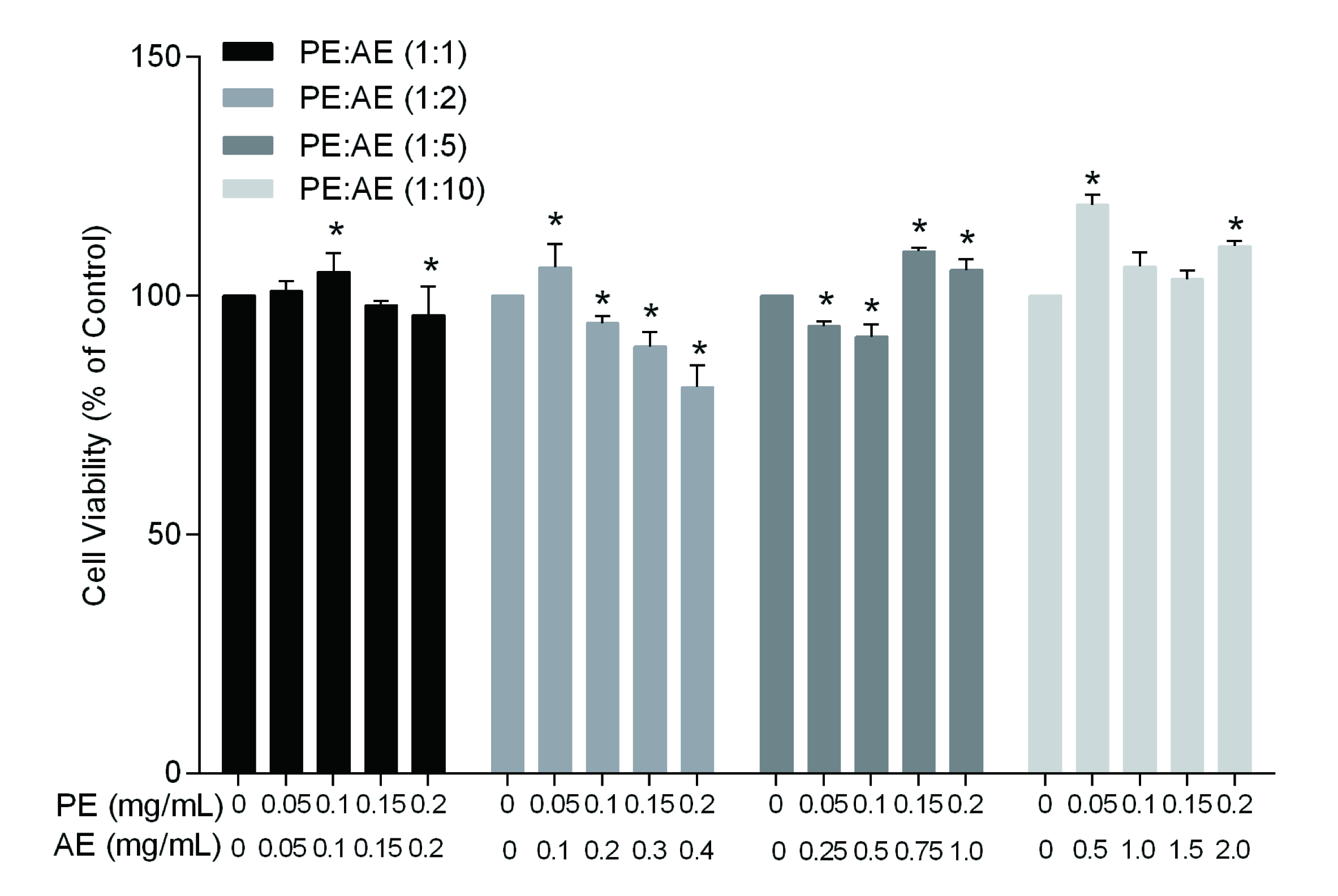

Supplement: Supplementary file 2 [file Image1.TIF]
