# Supplementary material for: Investigation of the mechanism of the anti-cancer effects of Astragalus propinquus Schischkin and Pinellia pedatisecta Schott (A&P) on melanoma via network pharmacology and experimental verification
Source: Front Pharmacol. 2022 Aug 12;13:895738. doi: 10.3389/fphar.2022.895738 (PMC9411814; doi:10.3389/fphar.2022.895738)
Supplement: Supplementary file 4 [file Table2.DOCX]

**Supplementary Table S2 ▏13 bioactive compounds of A&P from TMCSP and HPLC**

| **Molecule ID** | **Molecule name** | **Formula** | **OB(%)** | **DL** | **Structure** |
| --- | --- | --- | --- | --- | --- |
| MOL000098 | Quercetin | C_15_H_10_O_7_ | 46.43 | 0.28 | 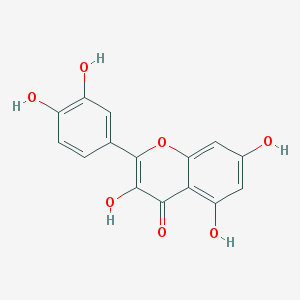 |
| MOL000296 | Hederagenin | C_29_H_50_O | 36.91 | 0.75 | 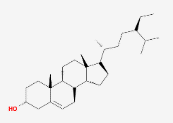 |
| MOL000354 | Isorhamnetin | C_16_H_12_O_7_ | 49.6 | 0.31 | 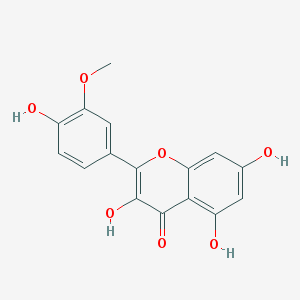 |
| MOL000387 | Bifendate | C_20_H_18_O_10_ | 31.1 | 0.67 | 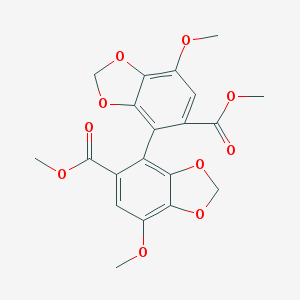 |
| MOL000392 | Formononetin | C_16_H_12_O_4_ | 69.67 | 0.21 | 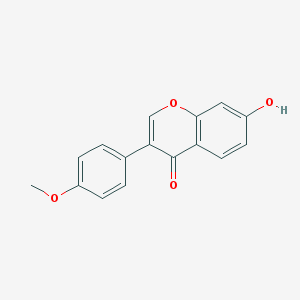 |
| MOL000417 | Calycosin | C_16_H_12_O_5_ | 47.75 | 0.24 | 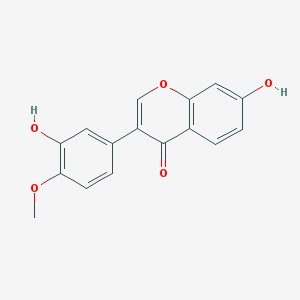 |
| MOL000422 | Kaempferol | C_15_H_10_O_6_ | 41.88 | 0.24 | 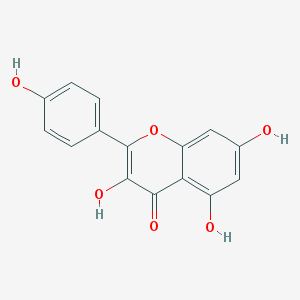 |
| MOL000415 | Rutin | C_27_H_30_O_16_ | 3.2 | 0.68 | 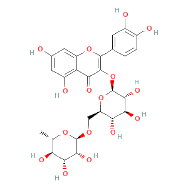 |
| MOL000431 | Coumarin | C_9_H_6_O_2_ | 29.17 | 0.04 | 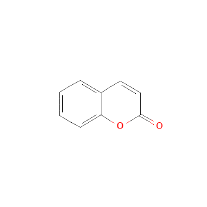 |
| MOL002714 | Baicalein | C_15_H_10_O_5_ | 33.52 | 0.21 | 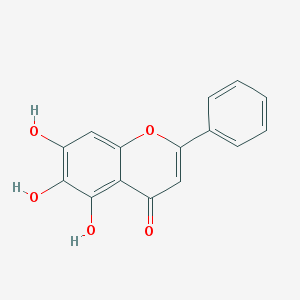 |
| MOL000358 | Beta-sitosterol | C_29_H_50_O | 36.91 | 0.75 | 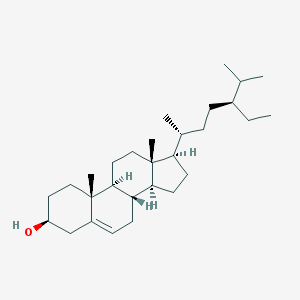 |
| MOL000449 | Stigmasterol | C_29_H_48_O | 43.83 | 0.76 | 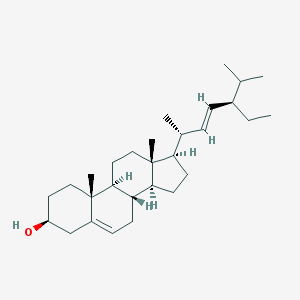 |
| MOL001452 | Protocatechualdehyde | C_7_H_6_O_3_ | 38.35 | 0.03 | 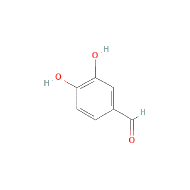 |
